# Supplementary material for: Ecological Effect of Differently Treated Wooden Materials on Microalgal Biofilm Formation in the Grado Lagoon (Northern Adriatic Sea)
Source: Microorganisms. 2023 Aug 30;11(9):2196. doi: 10.3390/microorganisms11092196 (PMC10537043; doi:10.3390/microorganisms11092196)
Supplement: Supplementary file 1 [file microorganisms-11-02196-s001.zip › Figures S1_S2.pdf]

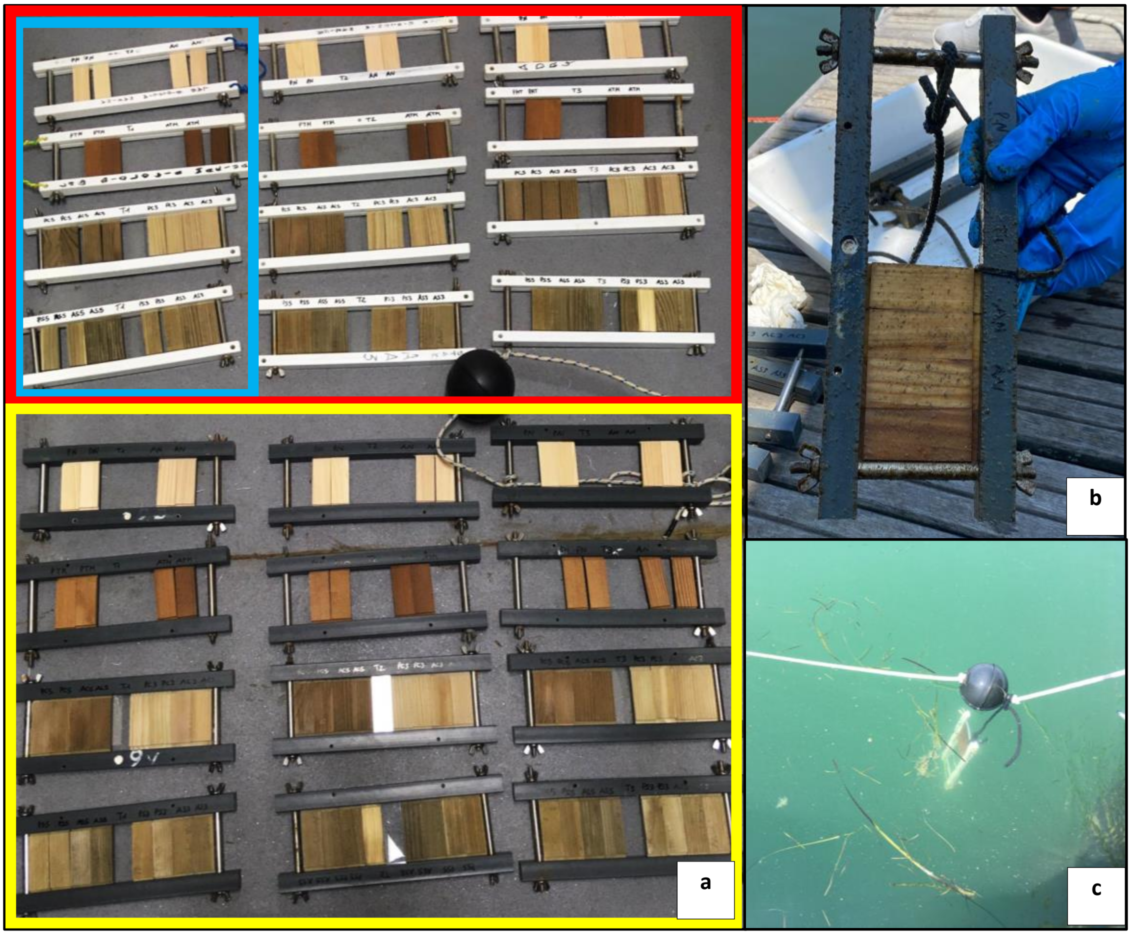

Figure S1: Series of collectors divided by area (a): 12 were immersed in the "Schiusa" area, highlighted by the red square and 12 were immersed in the "Approdo" area (yellow square). From each area, 4 collectors were retrieved at each experimental time (blue square). The collectors included two replicates of each treatment for each type of wood (b). Collectors were kept 50 cm from the water surface, regardless of the tidal range, using a system of buoys (c).

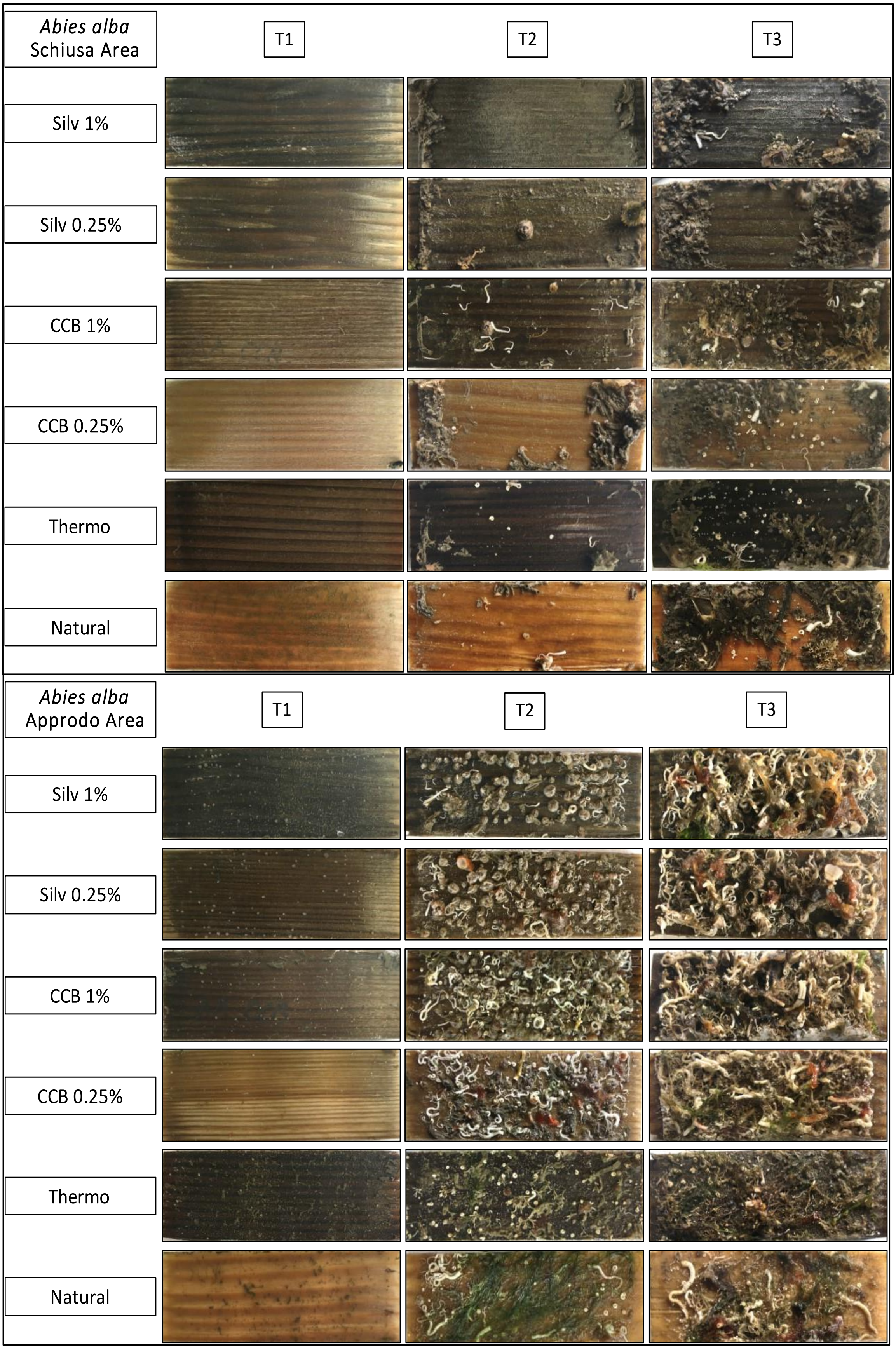

Figure S2: Photographs of diversely treated wooden panels (*Abies alba*) retrieved from the two areas of the Grado lagoon at three experimental times: after 6 days (T1), 20 days (T2) and 40 days (T3) of immersion.
